# Association between serum *β*-carotene and suicidal ideation in adults: a cross-sectional study

**DOI:** 10.3389/fnut.2024.1500107

**Published:** 2024-12-19

**Authors:** Jihua Lv, Tong Xu, Shuyue Lou, Zhenxiang Zhan, Zicheng Cheng, Fangwang Fu

**Affiliations:** ^1^Department of Internal Medicine, The Second People’s Hospital of Yongkang, Yongkang, China; ^2^Department of Neurology, The Second Affiliated Hospital and Yuying Children’s Hospital of Wenzhou Medical University, Wenzhou, China; ^3^Department of Neurology, Affiliated Jinhua Hospital, Zhejiang University School of Medicine, Jinhua, China

**Keywords:** *β*-carotene, carotenoid, depressive disorder, NHANES, suicidal ideation

## Abstract

**Background:**

The aim was to ascertain whether serum carotenoid levels are linked to suicidal ideation, along with how depressive disorder influences the association.

**Methods:**

This research was conducted using a cross-sectional design and encompassed 7,335 adults from the United States. The levels of serum carotenoids, encompassing *α*-carotene, *β*-carotene, *β*-cryptoxanthin, lycopene, and lutein/zeaxanthin, were ascertained by employing high-performance liquid chromatography. Item nine of the nine-item Patient Health Questionnaire was used to evaluate suicidal ideation. The study used multivariable-adjusted logistic regression and restricted cubic spline models to ascertain the link between serum carotenoid levels and suicidal ideation. Mediation and stratified analyses were used to ascertain the impact of depressed symptoms on the association between serum carotenoid levels and suicidal ideation.

**Results:**

Out of the total participants, 245 individuals (3.3%) reported having suicidal ideation. Participants who had suicidal ideation showed lower levels of serum *α*-carotene, *β*-carotene, *β*-cryptoxanthin, and lutein/zeaxanthin compared to those who did not have suicidal ideation. After controlling potential confounding factors, serum *β*-carotene level was still associated with risk of suicidal ideation (per 1–standard deviation (SD) increment, odds ratio [OR]: 0.73, 95% confidence interval [CI]: 0.55–0.98). The mediation analysis revealed that 36.3% of this association was mediated by the severity of depressive symptoms. Stratified analysis manifested that the association remained in depressed people but was attenuated in people without depressive disorder.

**Conclusion:**

Increased serum *β*-carotene level may decrease the susceptibility to suicidal ideation, especially in depressed people. Further intervention studies are needed to validate whether *β*-carotene consumption contributes to preventing suicidal ideation.

## Introduction

On a global scale, suicide is a significant public health concern, resulting in over 700,000 fatalities annually (1). The World Health Organization (WHO) has devised a strategy to decrease the number of suicide fatalities by 33% in each member state from 2013 to 2030 (2). Suicidal ideation is a significant factor that often leads to suicide attempts and completed suicides, according to ideation-to-action theories of suicide (3). It was estimated that the first-year risk of completed suicide after the expression of suicidal ideation was 1.40% in psychiatric patients (4). Therefore, identifying modifiable risk factors for suicidal ideation is beneficial for preventing suicide. The reported risk factors include sociodemographic characteristics, personality traits, and mood disorders (5, 6), but these factors are often unmodifiable.

Carotenoids are a group of essential pigments for human health that exert various physiological effects, including anti-inflammatory, antioxidant, immune modulation, intercellular communication, and provitamin A activity (7). The common and detectable carotenoids in the blood are *α*-carotene, *β*-carotene, *β*-cryptoxanthin, lycopene, and lutein/zeaxanthin. Literature shows carotenoid consumption to be associated with mental illnesses, including depression (8), anxiety disorder (9), and insomnia (10). Increasing evidence suggests that populations with lower serum carotenoid levels are more likely to develop depression (11, 12). Depression would lead to an increased risk of suicidal ideation (13); thus, it is plausible that serum carotenoids are associated with suicidal ideation.

Research data on serum carotenoid levels in suicidal ideation are extremely scarce at present. Therefore, we proposed to ascertain the correlation between serum carotenoids and suicidal ideation in a representative sample of the United States (US) adult population. We hypothesized that lower serum carotenoid levels might be linked to an elevated likelihood of suicidal ideation. Given that depressive symptoms significantly contribute to the likelihood of having suicidal ideation, we also investigated the effects of depressive disorder on the connection between serum carotenoids and suicidal ideation.

## Methods

### Study population

**Figure 1:**
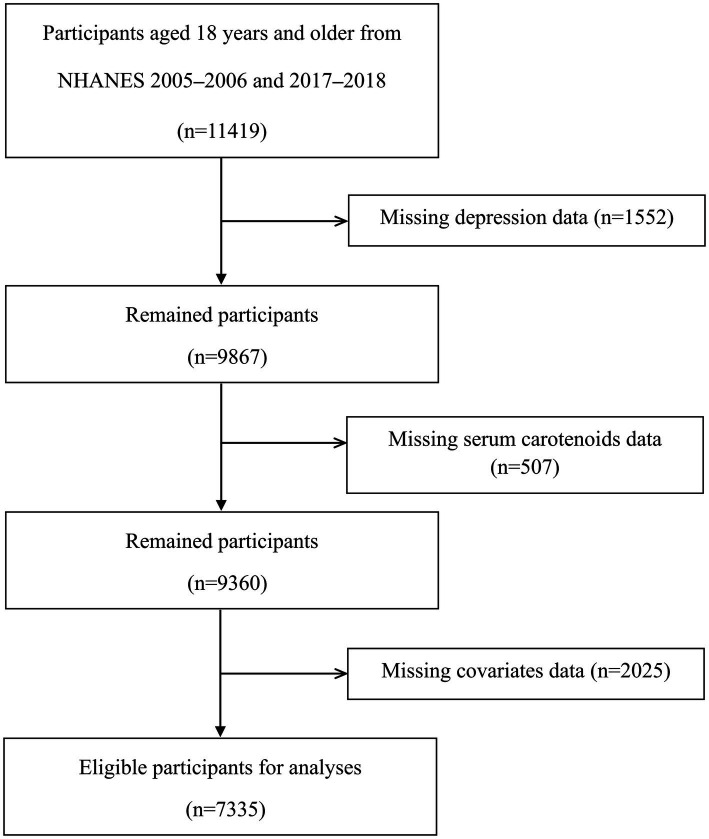
Flowchart of participant selection for this study.

This research employed a cross-sectional approach, and data were collected from the National Health and Nutrition Examination Survey (NHANES). NHANES is a biennial study program performed to ascertain the health and nutritional status of both adults and children in the US. A complex, multistage, probability sampling design was deployed to choose a representative sample of non-institutionalized individuals from the American population. Every participant had a standardized interview conducted at their home, followed by a physical examination, laboratory tests, and a second interview in a mobile examination center (MEC). The NHANES procedures were authorized by the Ethics Review Board of the National Center for Health Statistics, and participants signed to indicate that they had given informed consent. The original inclusion criteria for this study encompassed all participants from the NHANES 2005–2006 and 2017–2018 cycles who were 18 years of age or older, resulting in a total of 11,419 individuals. After excluding 1,552 participants who did not provide depressive disorder data assessed by the nine-item Patient Health Questionnaire (PHQ-9), 507 participants who did not provide serum carotenoid data, and 2025 participants who did not provide full covariate data, our study covered a total of 7,335 individuals with complete data available for statistical analysis (Figure 1).

### Serum carotenoids measurement

Fasting blood samples were collected in red-top or royal blue-top Vacutainer^®^ tubes, stored at −70°C, and shipped on dry ice to the National Center for Environmental Health for serum carotenoid measurement. The quantities of carotenoids in the serum, encompassing *α*-carotene, *β*-carotene, *β*-cryptoxanthin, lycopene, and lutein/zeaxanthin, were ascertained employing high-performance liquid chromatography (HPLC) with photodiode array absorbance detection. The minimum amounts that can be detected for *α*-carotene, *β*-carotene, *β*-cryptoxanthin, lycopene, and lutein/zeaxanthin are 0.5, 1.1, 0.6, 0.7, and 1.7 μg/dL, respectively. Participants with serum carotenoid levels below the detection limit were considered to have missing serum carotenoid data.

### Assessments of suicidal ideation and depressive disorder

The PHQ-9 is often employed to estimate patients’ mental well-being during the last 2 weeks (14). The scale has nine items, each of which is assessed and scored accordingly: 0 represents the absence of the condition, 1 indicates the presence of the condition for many days, 2 signifies the condition occurring for more than half the days, and 3 indicates the condition occurring nearly every day. Suicidal ideation was evaluated by analyzing the answer to item nine of the PHQ-9 questionnaire, which asked: “Over the last two weeks, how often have you felt that you would be better off dead, or hurting yourself in some way?.” Participants with a score of 1–3 were considered to have suicidal ideation, while those with a score of zero were considered to be without suicidal ideation. Removing item nine, the first eight questions (PHQ-8) were employed to compute a depression score (15). A cutpoint ≥10 was used to identify depressive symptoms. Participants with PHQ-8 score ≥ 10 or taking antidepressants are diagnosed with depressive disorder.

### Covariates

Covariates of interest consisting of sociodemographic, physical examination, and self-reported health data were deemed potential confounders based on previous studies (5, 6) and the variables that NHANES can provide. Trained interviewers gathered sociodemographic variables and self-reported health data (prior medical history, smoking and drinking status) during the household and MEC interviews. The sociodemographic variables were age, sex, race, education level, marital status, and the family income-to-poverty ratio. Trained health technicians gathered physical examination data, such as body mass index (BMI), waist circumference, and blood pressure in the MEC. Blood samples for the assessment of plasma fasting glucose, glycohemoglobin, total cholesterol, and low-density lipoprotein cholesterol (LDL-C) were collected in the MEC. Hypertension was determined by self-report, systolic blood pressure ≥ 140 mmHg, or diastolic blood pressure ≥ 90 mmHg. Diabetes mellitus was diagnosed by self-report, fasting blood glucose ≥7.0 mmol/L, or hemoglobin A1c ≥ 6.5%. The diagnosis of hyperlipidemia was based on self-report, total cholesterol ≥5.17 mmol/L, or LDL-C ≥ 3.37 mmol/L. The occurrence of coronary heart disease, stroke, and cancer was ascertained by employing self-reported diagnosis. Smoking history was characterized by the consumption of at least 100 cigarettes throughout the course of one’s lifetime. Drinking history is the practice of consuming at least 12 alcoholic drinks throughout a span of 1 year.

### Statistical analysis

The unweighted frequency and weighted percentage were utilized to represent categorical variables, whereas the weighted median and interquartile range were utilized to depict continuous data. The intergroup differences in continuous and categorical variables were compared using the Wilcoxon rank–sum test and the chi-squared test with Rao and Scott’s second-order correction, respectively. The serum carotenoid levels were considered as continuous variables and categorized into four quartiles as categorical variables. Logistic regression analysis was conducted to ascertain the connection between serum carotenoids and suicidal ideation. The Crude Model failed to include any potential confounding variables. Model 1 was controlled for age, gender, race, education level, marital status, and the family income-to-poverty ratio; Model 2 was controlled for covariates in Model 1 and body mass index, waist circumference, smoking, and drinking; Model 3 was controlled for covariates in Model 2 and hypertension, diabetes mellitus, hyperlipidemia, coronary heart disease, stroke, and cancer; Model 4 was controlled for covariates in Mode 3 and depressive disorder.

A restricted cubic spline regression model was employed to investigate the potential non-linear connection between serum carotenoids and suicidal ideation (16). The model included four knots located at the 5, 35, 65, and 95^th^ percentiles. The median of each serum carotenoid level was the reference point. Mediation analysis was employed to estimate the possible mediating implications of depressive disorder score on the link between serum *β*-carotene and suicidal ideation (17). Stratified analysis was conducted to ascertain the potential moderating effects of depressive disorder (yes/no). The above analyses were adjusted for the same covariates as in the Model 4. The statistical analyses were performed employing R version 4.2.1, which was created by the R Foundation for Statistical Computing in Vienna, Austria. A two-tailed *p*-value less than 0.05 was deemed to be statistically significant. Sample weights were used to illustrate the complex sampling design of the NHANES.

## Results

### Participants characteristics

Table 1 displays the fundamental features of the participants. Out of the 7,335 participants, 245 individuals (3.3%) were found to have suicidal ideation, whereas 1,120 individuals (16.7%) were diagnosed with depressive disorder. The median age was 46.0 years, with females accounting for 51.1% of the population. The majority of residents, namely 70.0%, were non-Hispanic White persons. The levels of *α*-carotene, *β*-carotene, *β*-cryptoxanthin, lycopene, and lutein/zeaxanthin in the serum were measured to be 3.2 (1.8–6.0), 12.8 (7.0–24.0), 6.9 (4.3–11.2), 40.2 (29.2–53.7), and 15.2 (10.6–21.9) μg/dL, respectively. Compared with those without suicidal ideation, participants with suicidal ideation were more likely to be non-White persons, living alone, and smokers. In addition, those with suicidal ideation had lower education and family income-to-poverty ratio levels. They also had higher PHQ-8 scores and were more likely to have diabetes mellitus, stroke, and depressive disorder. The levels of serum *α*-carotene, *β*-carotene, *β*-cryptoxanthin, and lutein/zeaxanthin were lower in the group with suicidal ideation (all *p* < 0.05), whereas the levels of serum lycopene were comparable between the groups with suicidal ideation and without suicidal ideation.

**Table 1:** Participants’ characteristics stratified by the presence of suicidal ideation.

	Overall (*N* = 7,335)	Non-suicidal ideation (*N* = 7,090)	Suicidal ideation (*N* = 245)	*p*-value
Age (years)	46.0 (33.0–59.4)	46.0 (33.0–60.0)	47.2 (34.0–59.0)	0.71
Sex				0.62
Female	3,709 (51.1%)	3,582 (51.1%)	127 (49.1%)	
Male	3,626 (48.9%)	3,508 (48.9%)	118 (50.9%)	
Race				<0.001
Mexican American	1,213 (8.0%)	1,167 (8.0%)	46 (8.6%)	
Other Hispanic	422 (4.6%)	387 (4.4%)	35 (12.6%)	
Non-Hispanic White	3,348 (70.0%)	3,258 (70.4%)	90 (58.5%)	
Non-Hispanic Black	1,626 (10.3%)	1,581 (10.3%)	45 (11.0%)	
Other Race	726 (7.0%)	697 (6.9%)	29 (9.3%)	
Education level				<0.001
Less than high school	1,559 (12.7%)	1,479 (12.5%)	80 (21.6%)	
High school	1,775 (26.0%)	1,709 (25.9%)	66 (28.6%)	
More than high school	4,001 (61.3%)	3,902 (61.6%)	99 (49.7%)	
Marital status				<0.001
Married/living with partner	4,578 (65.5%)	4,470 (66.0%)	108 (47.8%)	
Widowed/divorced/separated	1,576 (18.4%)	1,494 (18.0%)	82 (32.1%)	
Never married	1,181 (16.2%)	1,126 (16.0%)	55 (20.1%)	
Family income-to-poverty ratio	3.3 (1.7–5.0)	3.3 (1.7–5.0)	1.9 (1.0–3.3)	<0.001
Body mass index (kg/m^2^)	28.1 (24.4–32.9)	28.1 (24.4–32.9)	29.3 (24.6–34.2)	0.13
Waist circumference (cm)	98.2 (87.0–110.2)	98.1 (87.0–110.1)	100.1 (90.0–113.9)	0.14
Smoking	3,468 (47.4%)	3,316 (47.0%)	152 (60.1%)	0.006
Drinking	4,394 (65.3%)	4,262 (65.5%)	132 (59.6%)	0.16
Hypertension	2,926 (35.8%)	2,813 (35.6%)	113 (43.8%)	0.14
Diabetes mellitus	1,174 (11.8%)	1,126 (11.6%)	48 (18.3%)	0.024
Hyperlipidemia	4,247 (56.9%)	4,108 (56.8%)	139 (61.0%)	0.27
Coronary heart disease	305 (3.6%)	287 (3.5%)	18 (6.2%)	0.083
Stroke	275 (2.6%)	259 (2.4%)	16 (6.9%)	<0.001
Cancer	654 (9.2%)	627 (9.1%)	27 (11.8%)	0.25
Depressive disorder	1,120 (16.7%)	959 (15.1%)	161 (68.6%)	<0.001
PHQ-8 score	1.0 (0.0–4.0)	1.0 (0.0–4.0)	11.6 (6.0–15.0)	<0.001
α-carotene (μg/dL)	3.2 (1.8–6.0)	3.2 (1.8–6.1)	2.6 (1.4–4.6)	<0.001
β-carotene (μg/dL)	12.8 (7.0–24.0)	12.9 (7.1–24.1)	9.6 (5.4–19.9)	<0.001
β-cryptoxanthin (μg/dL)	6.9 (4.3–11.2)	7.0 (4.3–11.2)	5.9 (3.6–9.4)	0.010
Lycopene (μg/dL)	40.2 (29.2–53.7)	40.3 (29.4–53.6)	38.0 (23.0–56.0)	0.17
Lutein/zeaxanthin (μg/dL)	15.2 (10.6–21.9)	15.3 (10.7–22.0)	13.9 (9.1–20.5)	0.027

PHQ-8, eight-item Patient Health Questionnaire.

### Serum *β*-carotene and suicidal ideation

The logistic regression analysis findings for the connection between serum *β*-carotene and suicidal ideation are shown in Table 2. The unadjusted model indicated a reduced likelihood of having suicidal ideation for every 1-standard deviation (SD) increase in serum *β*-carotene level (odds ratio [OR]: 0.60, 95% confidence interval [CI]: 0.46–0.78, *p* < 0.001). Following the adjustment for sociodemographic variables (Model 1), serum *β*-carotene and suicidal ideation were still significantly associated (OR: 0.65, 95% CI: 0.50–0.84, *p* = 0.003). After further adjustment for body mass index, waist circumference, smoking, and drinking (Model 2), the association between serum *β*-carotene and suicidal ideation was not significantly changed (OR: 0.69, 95% CI: 0.53–0.90, *p* = 0.009). Even after further adjustment for all relevant comorbidities (Model 3), the significant association between serum *β*-carotene and suicidal ideation remained (OR: 0.69, 95% CI: 0.51–0.94, *p* = 0.023). Finally, depressive disorder was adjusted (Model 4) and the association remained stable (OR: 0.73, 95% CI: 0.55–0.98, *p* = 0.041).

**Table 2:** Logistic regression analysis to identify the association between serum β-carotene and suicidal ideation.

	Crude model		Model 1		Model 2		Model 3		Model 4	
	OR (95% CI)	*p*–value	OR (95% CI)	*p*–value	OR (95% CI)	*p*–value	OR (95% CI)	*p*–value	OR (95% CI)	*p*–value
Per 1–SD increase	0.60 (0.46–0.78)	<0.001	0.65 (0.50–0.84)	0.003	0.69 (0.53–0.90)	0.009	0.69 (0.51–0.94)	0.023	0.73 (0.55–0.98)	**0.041**
Q1 (≤6.9)	ref		ref		ref		ref		ref	
Q2 (7.0–13.0)	0.75 (0.54–1.03)	0.073	0.80 (0.55–1.16)	0.22	0.83 (0.56–1.23)	0.33	0.84 (0.55–1.29)	0.36	0.91 (0.52–1.58)	0.67
Q3 (13.1–24.0)	0.55 (0.38–0.79)	0.002	0.60 (0.39–0.92)	0.022	0.65 (0.41–1.03)	0.064	0.66 (0.39–1.12)	0.10	0.70 (0.38–1.29)	0.20
Q4 (≥24.1)	0.52 (0.28–0.94)	0.031	0.61 (0.32–1.15)	0.12	0.69 (0.35–1.37)	0.26	0.71 (0.32–1.55)	0.32	0.85 (0.37–1.96)	0.64
*P* for trend	0.010		0.055		0.16		0.21		0.46	

CI, confidence interval; OR, odds ratio; SD, standard deviation. Model 1: adjusted for age + sex + race + education level + marital status + family income-to-poverty ratio. Model 2: Model 1 + body mass index + waist circumference + smoking + drinking. Model 3: Model 2 + hypertension + diabetes mellitus + hyperlipidemia + coronary heart disease + stroke + cancer. Model 4: Model 3 + depressive disorder. The bold values represent statistically significant results (*p* < 0.05).

**Figure 2:**
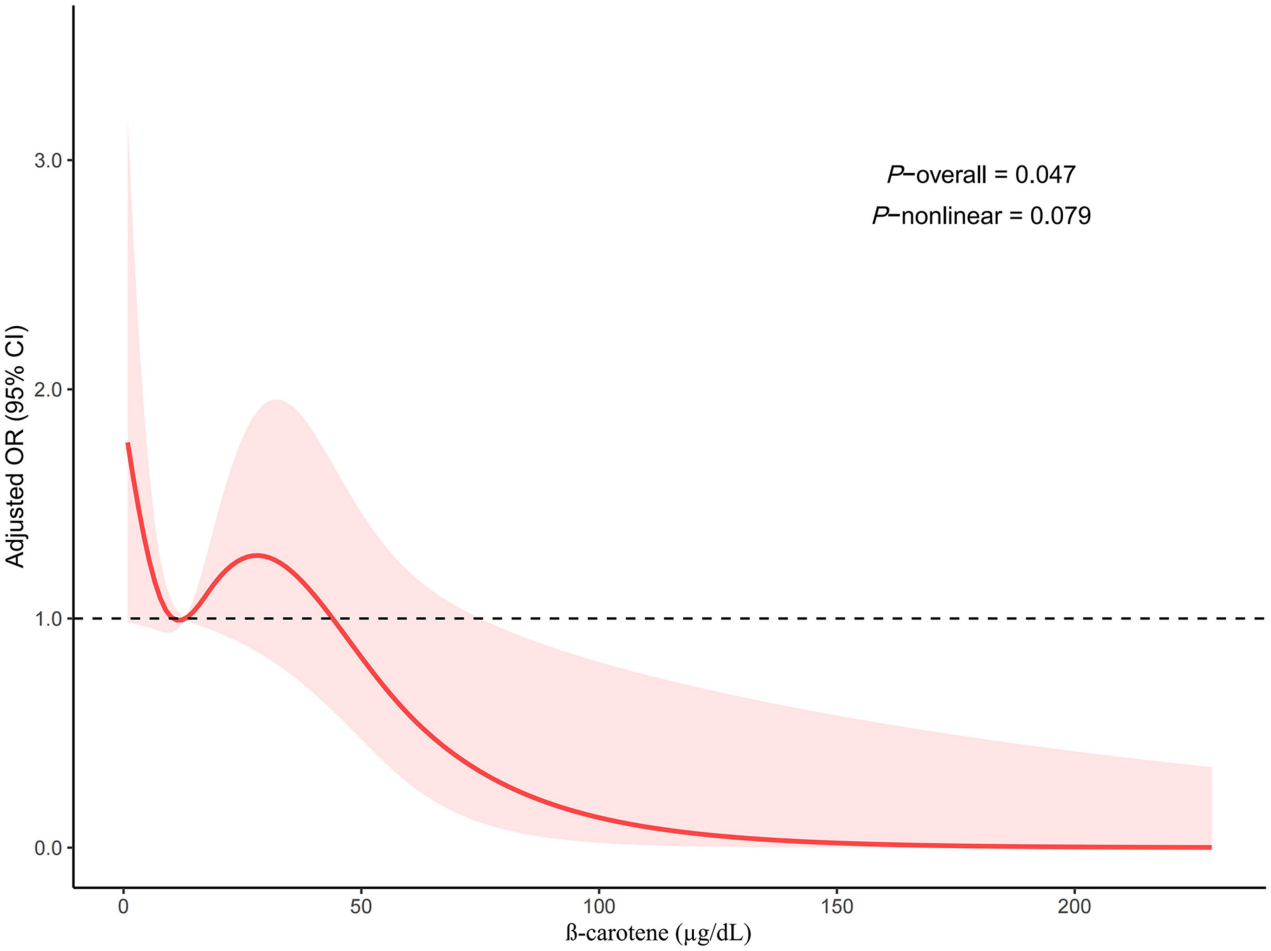
Restricted cubic spline regression model of the association between serum *β*-carotene level and the risk of suicidal ideation. CI, confidence interval; OR, odds ratio.

**Figure 3:**
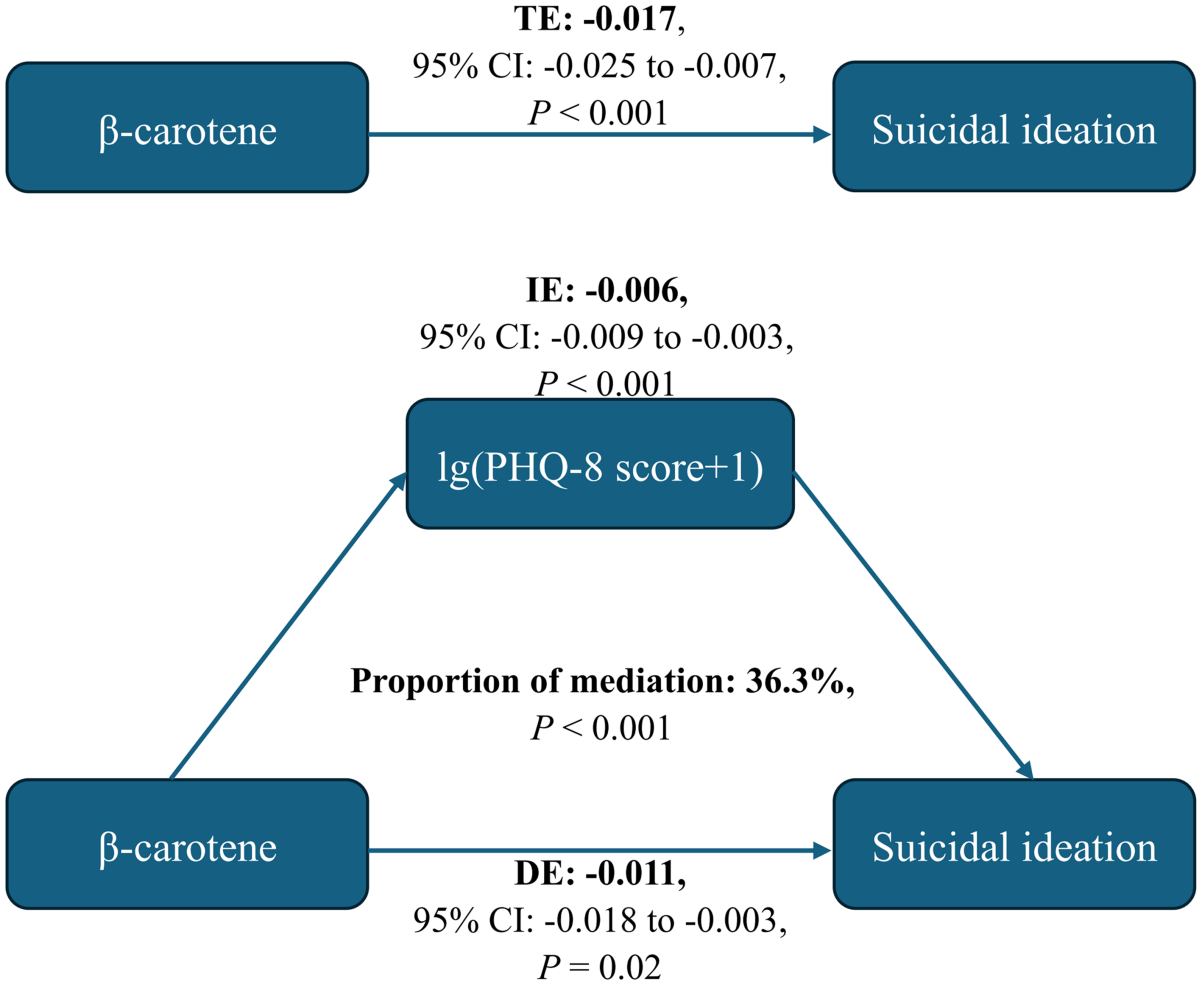
Mediating effects of depressive symptoms on the association between serum β-carotene level and the risk of suicidal ideation. CI, confidence interval; DE, direct effect; IE, indirect effect; PHQ, Patient Health Questionnaire; TE, total effect.

**Figure 4:**
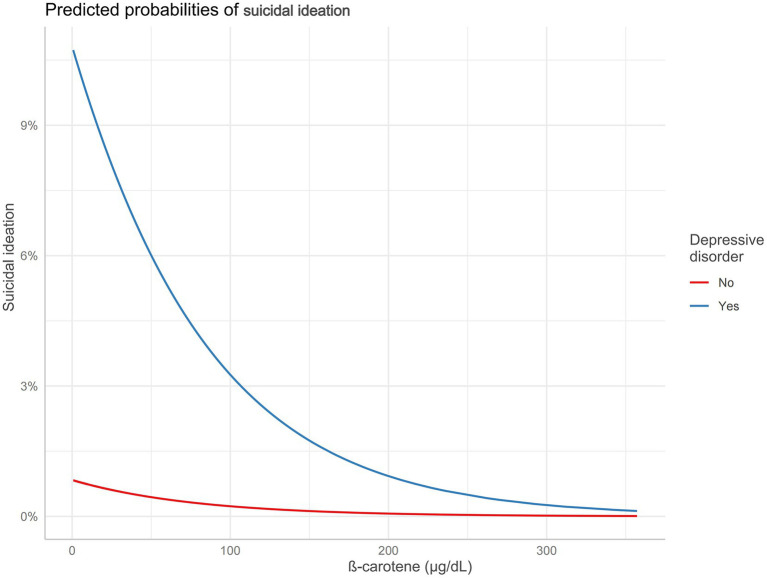
Predicted probabilities of suicidal ideation according to serum β-carotene level stratified by the presence of depressive disorder.

The restricted cubic spline regression model demonstrated that serum *β*-carotene level was inversely with risk of suicidal ideation in a linear manner (*p*-overall = 0.047*, p*-nonlinear = 0.079; Figure 2). As shown in Figure 3, the total effect represented the implication of serum *β*-carotene on suicidal ideation (*p* < 0.001); the direct effect represented the implication of serum *β*-carotene on suicidal ideation, not mediated by PHQ-8 score (*p* = 0.02); the indirect effect represented the implication of serum *β*-carotene on suicidal ideation, mediated by PHQ-8 score (*p* < 0.001). The proportion of PHQ-8 score mediating the effect of serum *β*-carotene on suicidal ideation was 36.3%. According to the results of stratified analysis by depressive disorder, serum *β*-carotene level was significantly associated with the probability of experiencing suicidal ideation in individuals with depressive disorder (OR: 0.62, 95% CI: 0.39–0.97, *p* = 0.039) rather than those free of depressive disorder (OR: 0.90, 95% CI: 0.69–1.17, *p* = 0.38). Figure 4 shows the reduced probability of suicidal ideation accompanied by incremental serum *β*-carotene level, especially in participants with depressive disorder.

### Other serum carotenoids and suicidal ideation

Although higher serum *α*-carotene, *β*-cryptoxanthin, lycopene, and lutein/zeaxanthin levels were linked to a lower risk of suicidal ideation in unadjusted or partially adjusted models, the association significantly attenuated in fully adjusted models incorporating depressive disorder (all *p* > 0.05; Supplementary Table S1). The restricted cubic spline regression model further showed no linear or non-linear relationship between other serum carotenoids and suicidal ideation (Supplementary Figure S1).

## Discussion

Within this sample of the adult population in the US, we saw lower levels of serum *β*-carotene in persons who had suicidal ideation compared to those who did not. The connection between serum *β*-carotene and suicidal ideation was independent of sociodemographic characteristics, health factors, comorbidities, and depressive disorder. The connection between serum *β*-carotene levels and the risk of suicidal ideation was somewhat mediated by the intensity of depressive symptoms, accounting for about one-third of the link. Depressed persons who had greater levels of serum *β*-carotene had a decreased likelihood of experiencing suicidal ideation. However, this relationship was weakened in those who did not have depressive disorder.

This analysis represents the first known study to explore the connection between serum carotenoids and suicidal ideation in the whole population. Carotenoids are natural pigments mainly sourced from fruits and vegetables. Consumption of fruits and vegetables more than one time per day can reduce the risk of suicidal ideation by 15% in the Korean population (18). The beneficial effects of fruits and vegetables may be ascribed to their rich content of antioxidant nutrients, including carotenoids. By calculating carotene intake according to the intake frequency and quantity of 112 foods, Lim et al. established a significant inverse association between the consumption of carotene and the likelihood of experiencing suicidal ideation (19). While there has been no prior research specifically investigating the connection between serum carotenoids and suicidal ideation, a previous study examined the association between serum carotenoids and suicide attempts. This investigation found that the probability of having low serum concentrations of total carotenoids was approximately twice as much in individuals with suicide attempts compared with those without (20). Overall, the current evidence provides insight that the intake of carotene has the potential to prevent suicidal ideation.

Inflammation and oxidative stress are involved in the pathophysiology of suicidal ideation. A recent comprehensive meta-analysis showed increased levels of blood immune-inflammatory and nitro-oxidative biomarkers (e.g., interleukin-6, tumor necrosis factor-*α*, interferon-*γ*, C-reactive protein, malondialdehyde) in individuals who had suicidal ideation versus controls (21). These biomarkers have neurotoxic effects and may cause brain dysfunctions. Emerging evidence suggests structural and functional alterations in frontal, limbic, and temporal lobes in suicidal ideation. Structural abnormalities include reduced gray matter volume and cortical thickness, while functional abnormalities include decreased fractional anisotropy and structural connectivity, increased radial diffusivity, and changed functional connectivity in a brain region associated with deficits in emotional processing and regulation (22). Carotenoids are powerful scavengers of reactive oxygen species (ROS), protecting neuronal cells from oxidative damage. By reducing ROS, carotenoids mitigate oxidative stress and preserve cellular integrity, potentially enhancing neurotransmitter balance and improving mood regulation (23). In addition to their antioxidant effects, carotenoids modulate key inflammatory pathways. Studies indicate that carotenoids can inhibit nuclear factor-kappa B (NF-κB) and downregulate pro-inflammatory cytokines such as interleukin-6 and tumor necrosis factor-*α*, reducing neuroinflammation associated with mood disorders (24). By curbing inflammation in neural tissues, carotenoids may help stabilize mood and lessen psychological distress.

Depression is closely related to suicidal ideation. Manifestations of major depressive disorder (MDD), such as profound feelings of despair or guilt and disruptions in sleep patterns, heighten the likelihood of suicidal tendencies (25). Compared to non-MDD controls, the ORs for lifetime and past-month prevalence of suicidal ideation in MDD were 2.88 and 49.88, respectively (26). In the meta-analysis by Dong et al., the lifetime and past-month prevalence of suicidal ideation in MDD were 53.1 and 27.7%, respectively (27). A moderate sample study found that higher plasma total carotenoid levels predicted a lower probability of depressed mood at a six-year follow-up (12). Another recent moderate sample study also indicated that a higher plasma total carotenoids/lipids ratio was linked to a mitigated risk of depressed mood over a median follow-up period of 13.4 years (11). By improving depressive symptoms, carotenoids indirectly hinder the risk of suicidal ideation.

In the multivariable model, when without accounting for depressive disorder, higher levels of serum *α*-carotene, *β*-carotene, lycopene, and lutein/zeaxanthin are linked to a decreased likelihood of experiencing suicidal ideation. After further considering the impact of depressive disorder, only serum *β*-carotene was linked to suicidal ideation in our investigation. This indicates that the protective effects of α-carotene, lycopene, and lutein/zeaxanthin on suicidal ideation are entirely mediated by depressive symptoms. By contrast, the mediating effect of depressive symptoms merely accounts for approximately one-third of the connection between serum *β*-carotene and suicidal ideation. On the one hand, depressive disorder is an important risk factor for suicidal ideation. On the other hand, depressive disorder and suicidal ideation share common risk factors and pathogenesis (i.e., inflammation and oxidative stress). Thus, the improvement of depressive disorder by carotenoids is usually accompanied by a reduction in the risk of suicidal ideation. However, depressive disorder only mediates partial association between serum *β*-carotene and suicidal ideation, indicating that there are residual mechanisms of protection of *β*-carotene worth studying.

It is important to acknowledge a few limitations before interpreting the findings. First, the cross-sectional design of this analysis prevents the establishment of a causal relationship. The relationship between serum *β*-carotene and suicidal ideation is probably reciprocal. The low serum *β*-carotene status among participants with suicidal ideation may be caused by insufficient intake sources or high inflammatory consumption. The mediation analysis relies on cross-sectional data, also limiting inference on the temporal sequence of mediation. The next step is to design a cohort study to observe how baseline levels or trajectories of serum *β*-carotene affect the risk of depression and suicidal ideation in the future, so as to clarify the causal link. Second, although the item nine of the PHQ-9 has been previously used to define suicide ideation, it includes non-suicidal harm, which may mistakenly estimate the strength of the association between serum carotenoids and suicidal ideation and the mediating effect of depressive disorder. The item nine of the PHQ-9 also fails to distinguish passive and active ideation. Without distinguishing the nuances of suicidal ideation, the applicability of findings to interventions may be limited. Third, limited by the data provided by NHANES, the psychiatric history of participants was unknown and may cause a significant bias in the current results. Fourth, carotenoids are obtained by consuming fruits and vegetables. This research did not investigate the correlation between the consumption of carotenoids in the diet and the occurrence of suicidal ideation. Future investigations are necessary to determine if increasing carotenoids consumption reduces the risk of suicidal ideation. Fifth, there may still be some remaining confounding effects that cannot be totally eliminated. For example, participants with high serum *β*-carotene levels tend to have healthier dietary patterns and more physical activity, which are associated with a reduced risk of suicidal ideation (28).

## Conclusion

Despite its limitations, our study first reported the independent association between serum *β*-carotene and suicidal ideation, which may introduce new therapeutic strategies for reducing the risk of suicidal ideation. We found that serum *β*-carotene is significantly associated with suicidal ideation only in participants with depressive disorder, not in those without depressive disorder. The relationship between serum *β*-carotene and suicidal ideation was partially mediated by depressed symptoms, which is worth mentioning. Further investigation is required before providing any definitive or authoritative suggestions for the use of *β*-carotene in preventing suicidal ideation.

## Data Availability

The raw data supporting the conclusions of this article will be made available by the authors, without undue reservation.
