# Supplementary material for: Association between serum β-carotene and suicidal ideation in adults: a cross-sectional study
Source: Front Nutr. 2024 Dec 19;11:1500107. doi: 10.3389/fnut.2024.1500107 (PMC11693589; doi:10.3389/fnut.2024.1500107)
Supplement: Supplementary file 1 [file Table_1.docx]

**Association between Serum β-Carotene and Suicidal Ideation in Adults: A Cross-sectional Study**

Table S1. Logistic regression analysis to identify the association between other serum carotenoids and suicidal ideation.

Figure S1. Restricted cubic spline regression model of the association between other serum carotenoid levels and the risk of suicidal ideation. CI, confidence interval; OR, odds ratio.

**Table S1** Logistic regression analysis to identify the association between other serum carotenoids and suicidal ideation.

|  | **Crude Model** | | **Model 1** | | **Model 2** | | **Model 3** | | **Model 4** | |
| --- | --- | --- | --- | --- | --- | --- | --- | --- | --- | --- |
|  | **OR (95% CI)** | ***P*–value** | **OR (95% CI)** | ***P*–value** | **OR (95% CI)** | ***P*–value** | **OR (95% CI)** | ***P*–value** | **OR (95% CI)** | ***P*–value** |
| **α-carotene (μg/dL)** | | | | | | | | | | |
| Per 1–SD increase | 0.73 (0.56–0.96) | 0.027 | 0.77 (0.59–1.00) | 0.049 | 0.82 (0.62–1.08) | 0.15 | 0.83 (0.61–1.13) | 0.20 | 0.84 (0.62–1.14) | 0.23 |
| Q1 (≤1.7) | ref |  | ref |  | ref |  | ref |  | ref |  |
| Q2 (1.8–3.2) | 0.61 (0.36–1.02) | 0.060 | 0.66 (0.37–1.17) | 0.15 | 0.69 (0.39–1.21) | 0.17 | 0.70 (0.38–1.29) | 0.20 | 0.74 (0.39–1.42) | 0.29 |
| Q3 (3.3–6.0) | 0.75 (0.51–1.11) | 0.15 | 0.81 (0.51–1.28) | 0.34 | 0.90 (0.55–1.46) | 0.63 | 0.91 (0.53–1.59) | 0.71 | 1.01 (0.56–1.81) | 0.97 |
| Q4 (≥6.1) | 0.44 (0.29–0.66) | <0.001 | 0.48 (0.31–0.74) | 0.003 | 0.55 (0.33–0.89) | 0.020 | 0.56 (0.32–0.99) | 0.047 | 0.65 (0.34–1.26) | 0.16 |
| *P* for trend | 0.002 |  | 0.012 |  | 0.077 |  | 0.13 |  | 0.35 |  |
| **β-cryptoxanthin (μg/dL)** | | | | | | | | | | |
| Per 1–SD increase | 0.79 (0.62–1.01) | 0.062 | 0.79 (0.61–1.03) | 0.073 | 0.85 (0.65–1.09) | 0.18 | 0.86 (0.65–1.13) | 0.24 | 0.99 (0.77–1.27) | 0.91 |
| Q1 (≤4.5) | ref |  | ref |  | ref |  | ref |  | ref |  |
| Q2 (4.6–7.6) | 0.61 (0.37–1.01) | 0.055 | 0.65 (0.38–1.13) | 0.12 | 0.68 (0.38–1.22) | 0.18 | 0.69 (0.36–1.31) | 0.21 | 0.82 (0.42–1.63) | 0.50 |
| Q3 (7.7–13.0) | 0.65 (0.43–0.99) | 0.044 | 0.69 (0.43–1.11) | 0.12 | 0.75 (0.47–1.19) | 0.20 | 0.77 (0.46–1.28) | 0.25 | 1.04 (0.58–1.88) | 0.88 |
| Q4 (≥13.1) | 0.60 (0.36–0.98) | 0.043 | 0.61 (0.34–1.09) | 0.088 | 0.71 (0.37–1.35) | 0.26 | 0.72 (0.34–1.55) | 0.34 | 1.01 (0.45–2.27) | 0.97 |
| *P* for trend | 0.048 |  | 0.099 |  | 0.30 |  | 0.39 |  | 0.79 |  |
| **Lycopene (μg/dL)** | | | | | | | | | | |
| Per 1–SD increase | 0.88 (0.71–1.10) | 0.26 | 0.95 (0.76–1.19) | 0.66 | 0.97 (0.78–1.20) | 0.75 | 0.96 (0.76–1.22) | 0.71 | 0.97 (0.75–1.26) | 0.79 |
| Q1 (≤27.0) | ref |  | ref |  | ref |  | ref |  | ref |  |
| Q2 (27.1–38.6) | 0.48 (0.31–0.76) | 0.003 | 0.55 (0.34–0.89) | 0.018 | 0.55 (0.34–0.89) | 0.020 | 0.57 (0.32–0.99) | 0.047 | 0.55 (0.30–1.02) | 0.056 |
| Q3 (38.7–52.6) | 0.52 (0.35–0.76) | 0.001 | 0.61 (0.40–0.95) | 0.029 | 0.63 (0.40–0.99) | 0.044 | 0.63 (0.38–1.06) | 0.075 | 0.63 (0.33–1.20) | 0.13 |
| Q4 (≥52.7) | 0.69 (0.40–1.21) | 0.19 | 0.87 (0.47–1.62) | 0.65 | 0.91 (0.50–1.67) | 0.74 | 0.90 (0.44–1.85) | 0.74 | 0.89 (0.40–1.98) | 0.72 |
| *P* for trend | 0.18 |  | 0.73 |  | 0.85 |  | 0.83 |  | 0.83 |  |
| **Lutein/zeaxanthin (μg/dL)** | | | | | | | | | | |
| Per 1–SD increase | 0.80 (0.63–1.03) | 0.077 | 0.83 (0.65–1.06) | 0.13 | 0.87 (0.68–1.11) | 0.25 | 0.87 (0.67–1.14) | 0.26 | 1.03 (0.80–1.33) | 0.79 |
| Q1 (≤11.3) | ref |  | ref |  | ref |  | ref |  | ref |  |
| Q2 (11.4–16.2) | 0.71 (0.46–1.11) | 0.12 | 0.73 (0.44–1.21) | 0.21 | 0.77 (0.46–1.27) | 0.27 | 0.77 (0.44–1.34) | 0.29 | 0.90 (0.47–1.71) | 0.68 |
| Q3 (16.3–22.9) | 0.49 (0.35–0.69) | <0.001 | 0.52 (0.35–0.75) | 0.002 | 0.55 (0.37–0.83) | 0.008 | 0.55 (0.35–0.86) | 0.017 | 0.77 (0.46–1.27) | 0.23 |
| Q4 (≥23.0) | 0.66 (0.37–1.19) | 0.16 | 0.72 (0.37–1.40) | 0.31 | 0.80 (0.39–1.64) | 0.51 | 0.79 (0.35–1.78) | 0.51 | 1.14 (0.46–2.86) | 0.72 |
| *P* for trend | 0.081 |  | 0.18 |  | 0.35 |  | 0.35 |  | 0.83 |  |

CI, confidence interval; OR, odds ratio; SD, standard deviation.

Model 1: adjusted for age + sex + race + education level + marital status + family income-to-poverty ratio.

Model 2: Model 1 + body mass index + waist circumference + smoking + drinking.

Model 3: Model 2 + hypertension + diabetes mellitus + hyperlipidemia + coronary heart disease + stroke + cancer.

Model 4: Model 3 + depressive disorder.


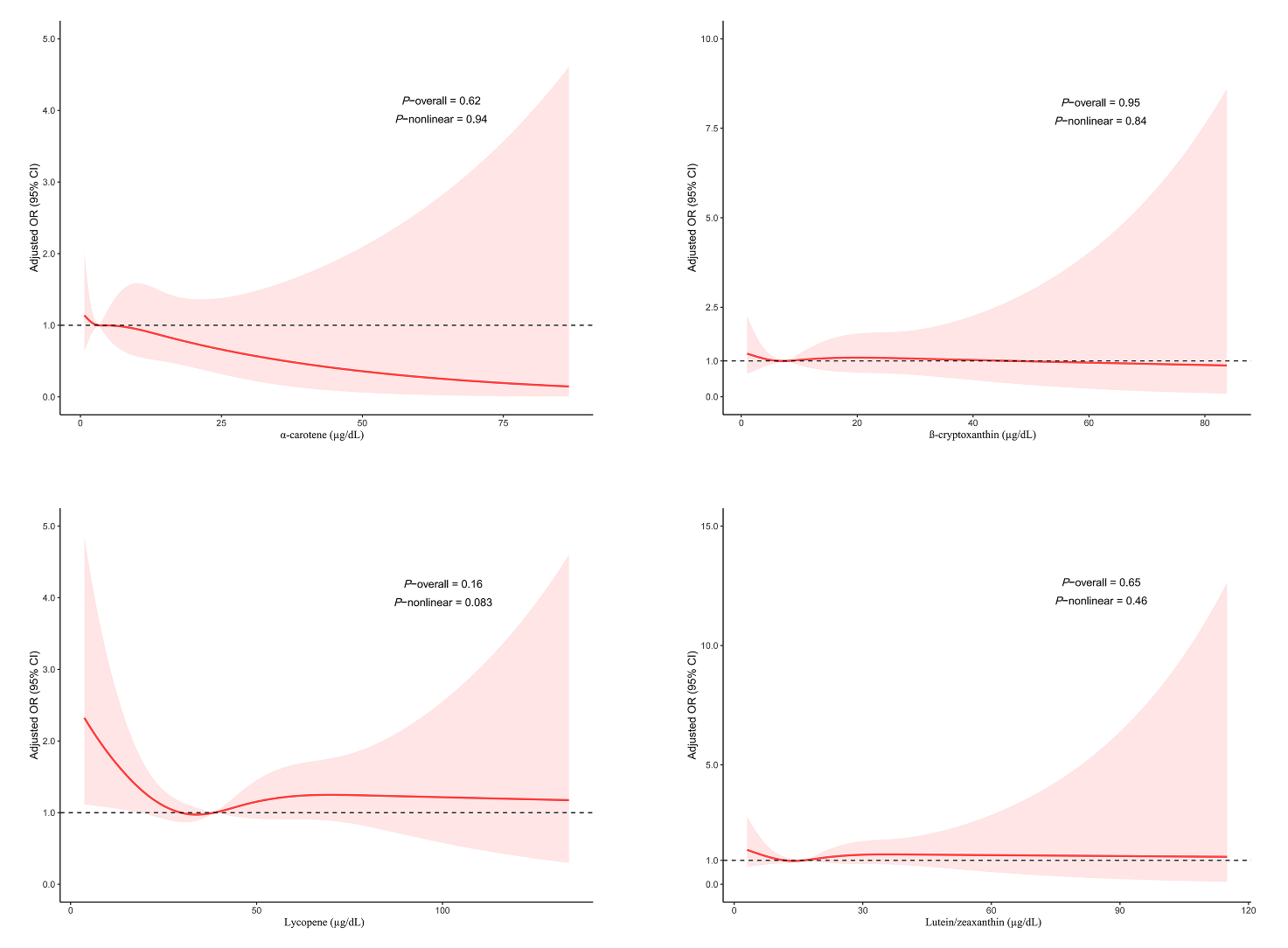


Figure S1. Restricted cubic spline regression model of the association between other serum carotenoid levels and the risk of suicidal ideation. CI, confidence interval; OR, odds ratio.
